# Removal of Pollution by Intensive Aeration Technology for Landfill Leachate Treatment

**DOI:** 10.5696/2156-9614-10.28.201212

**Published:** 2020-12-07

**Authors:** Hamri Zineb, Mouhir Latifa, Souabi Salah, Saafadi Laila

**Affiliations:** Faculty of Sciences and Techniques of Mohammedia, Hassan II University, Morocco

**Keywords:** leachate, aeration, landfill, pollution, waste

## Abstract

**Background.:**

Landfill leachate is a source of environmental pollution and a major concern for human health because it contains high concentrations of organic and inorganic contaminants.

**Objectives.:**

The objective of the present study is to validate the efficiency of the forced aeration treatment technique, which consists of intensively injecting a continuous oxygen flow of 16.75 kg/m^3^/h for 30 days using a bubble air diffuser in a relatively small volume of 1 m^3^. The principle of the technique is essentially based on acceleration of the degradation of the effluent as well as reduction of the organic and nitrogenous matter contents.

**Methods.:**

Forced aeration technology was used for the treatment of leachate from the Mohammedia-Benslimane landfill. The sample was treated by injecting a flow of oxygen to accelerate the biodegradability of the pollutants by the microorganisms.

**Results.:**

The physicochemical characterization of the raw leachate at the inlet of the aeration tank showed high values for chemical oxygen demand (COD) (38,600 mg O_2_/l), biological oxygen demand (BOD5) (24,000 mg O_2_/l), and total Kjeldahl nitrogen (TKN) (5,932.45 mg/L). The proposed treatment technique allowed relatively high purification yields to be achieved, with abatement rates for the major elements COD, BOD5 and TKN of 73%, 98%, and 85%, respectively.

**Conclusions.:**

The treatment of leachate by intensive aeration technology reduces considerably the pollutant load and achieves a high purification yield.

**Competing Interests.:**

The authors declare no competing financial interests.

## Introduction

Improved living standards, continued growth in industrial production and trade, and increased consumption have led to an increase in the volume of municipal and industrial waste globally.^1^ This has made waste management a challenge for countries around the world.^2^ In Morocco, about 0.78 kilos of waste is produced per day per capita in urban areas and 0.3 kilos per day per capita in rural areas.^2^

Morocco has not escaped this trend in the growth in the amount of waste produced, which has reached significant rates. By 2030, the total waste deposit estimated in 2015 at 26.8 million tons is projected to increase significantly to 39 million tons with an increase of 45%.^3^ The rate of waste production varies according to regions and can vary from 0.6 to 0.7 kg/person/day.^4^

This increase has repercussions for the management and landfilling of waste, leading to a saturation of landfills that are designed for both storage and treatment of waste. A large part of the potential risks associated with the landfilling of waste results from leachate migration and the release of noxious gases. As a result, landfills become a source of pollution for soil, air and human health rather than a solution for waste disposal.^5^

Leachate is the liquid waste produced by the percolation of meteoric precipitation water and runoff to which is added the water brought by the waste itself.^6^ It is an aqueous effluent with a complex and toxic composition that represents a threat to the environment, and its physico-chemical characteristics vary in time and space and depend on the nature of the waste. During the evolution of waste in landfills, the leachate becomes enriched with compounds that are not very biodegradable and its treatment becomes increasingly difficult with low purification yields.^7^

A number of measures have been taken by the Moroccan government to reduce the health and environmental impacts of waste, including implementation of a national strategy for the reduction and recovery of waste, establishment of a regulatory framework with the promulgation of Law 28-00 on waste management and disposal,^8^ implementation of a national programs for household and similar waste and establishment of management master plans in the provinces.

There are various leachate treatment technologies, including coagulation-flocculation, adsorption by activated carbon, biological treatment and membrane separation processes, such as nanofiltration and reverse osmosis (RO).^9^ Treatment efficiency depends on the operating conditions and the composition of the leachate in its initial state. Elimination of pollutants implies reduction of the pollutant load in order to reach the discharge standards imposed by Moroccan regulations.^10^

A novel treatment process is “intensive aeration technology”. Aeration systems have two main objectives: to provide aerobic micro-organisms with the oxygen, generally borrowed from the air, which they need to degrade organic matter, and to induce homogenization and mixing sufficient to ensure close contact between the living environment, polluting elements and oxygenated water.

The objective of the present study is to examine the efficiency and possibility of using intensive aeration technology for the treatment of leachate from the Mohammedia-Benslimane public landfill, Morocco by forced aeration at the national level, in order to assure a satisfactory pollutant load elimination rate and purification efficiency.

## Methods

**Figure 1:**
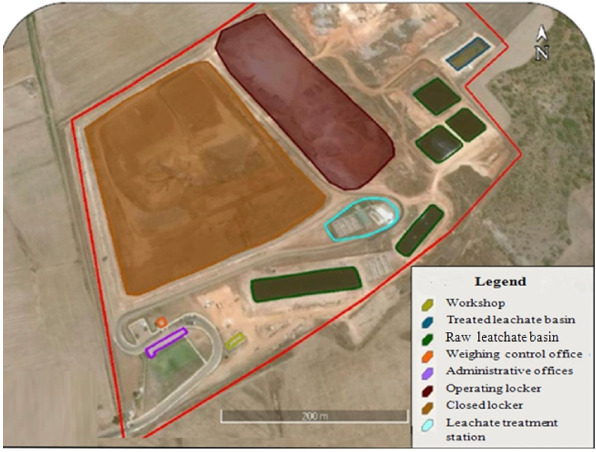
Mohammedia-Benslimane landfill site

The controlled landfill of Mohammedia-Benslimane is an interprovincial landfill intended to receive the waste of the prefecture of Mohammedia and the prefecture of Benslimane. It was created following the closure of the former Mesbahiat wild dump and occupies an area of 47 hectares *(Figure 1).* The aim of this work on the Mohammedia-Benslimane public landfill site was to continue studies and tests on the treatment of discharges from the prefectures served by the site using different techniques.

**Figure 2:**
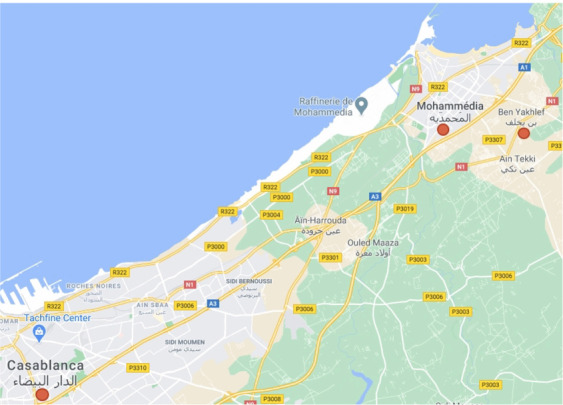
Map of study area

**Abbreviations**
*AFNOR*: Association Française de Normalisation (French Standardization Association)
*BOD5*: Biological oxygen demand
*COD*: Chemical oxygen demand
*EC*: Electrical conductivity

The Mohammedia-Benslimane landfill center is located about 8 km southeast of the center of Ben Yakhlef, 17 km east of the center of Mohammedia, 24 km southwest of the center of Benslimane, and 800 m from the provincial road RP 3313 precisely at Douar Beni M'ghit, on the edge of the left bank of Chaaba El Hamra, a tributary of the Oued Nfifikh.

The landfill is located on a site designed to accommodate five large traps (3 engineered and 2 projected with leachate and contaminated storm water drainage systems), five raw leachate ponds, one treated leachate pond, and one contaminated storm water pond. The site also contains a weighbridge to control waste intake and weighing and a bio gas drainage network for the closed bins is installed. There are also administrative offices, a weighing control office, room for a caretaker, and a maintenance workshop and repair. It receives an average waste flow of 564.78 tons/day and an average amount of leachate of 70 m^3^/day recovered in storage tanks. The study concerns all the communes of the prefecture of Mohammedia, including Mohammadia, Ain Harrouda, Bni Yakhlef, Ech-Chellalte, Sidi Moussa Ben Ali, and Sidi Moussa Majdoub.

**Figure 3:**
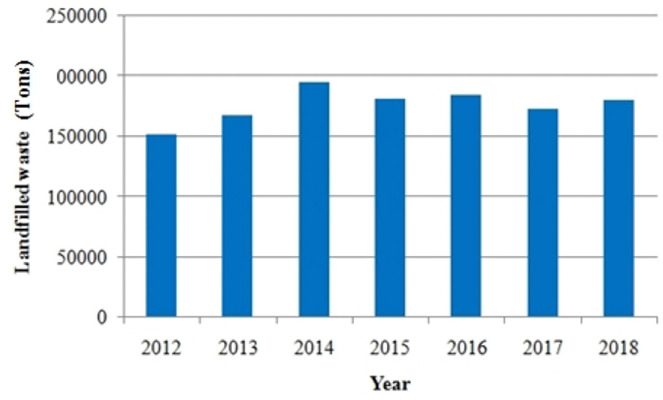
Quantity of waste buried in the Mohammedia-Benslimane landfill site

The quantities of waste produced are essentially based on the evolution of the population and the ratios of waste production per inhabitant. The values of the quantities of waste produced for each commune are represented in Table 1. The amount of waste received increased from approximately 151,501 tons in 2012 to 178,033 tons in 2018. Figure 3 illustrates the change in the amount of waste landfilled since the first year of landfill operation.

**Table 1:** Estimated Evolution of the Quantities of Household Waste Produced (tons/day)

**Population center**	**2015**	**2020**	**2030**
Mohammedia	189.6	190.5	192.4
Ain Harrouda	43.3	53.1	80.2+210
Bni Yakhlef	39.3	55.4	110.0
Ech-Chellalte	42.4	52.6	80.85
Sidi Moussa Ben Ali	7.5	8.21	9.91
Sidi Moussa Majdoub	10.6	11.5	13.8

## Landfill leachate management

The leachate is collected from the traps by a collection and drainage system located at the bottom of the traps, then transported by gravity through pipes connected to the main collector attached to a storage tank with a capacity of 30,000 m^3^. A tank with a volume of 50 m^3^ is filled with raw leachate to supply the treatment station.

The leachate is treated in the landfill by two methods: biological and physico-chemical. The physico-chemical treatment is characterized by addition of calcium oxide or lime to promote settling, addition of the coagulant ‘ferrous chloride' to settle the colloids, and addition of sulphuric acid and hydrogen peroxide to reduce their pollutant load. The biological treatment is based on filtration of the effluents by a sand filter planted with reeds in order to capture the residual pollutants in the leachate.

## Leachate sampling

In the study site, snapshot sampling was carried out weekly to monitor treatment progress over time. Samples were taken weekly for one day each week at a rate of 2 L/h for 8 hours. Leachate samples were collected in non-contaminating polyethylene vials, labeled with the date of collection and sample name, and transported to the laboratory at 4°C. The samples were collected from the leachate in a sealed, non-contaminating polyethylene vial.

The first raw sample was taken from the leachate storage tank to identify its initial characteristics, with the first sample representing the study reference. Then, further samples were taken from the reactor during the treatment period.

## Experimental method of aeration treatment

**Figure 4:**
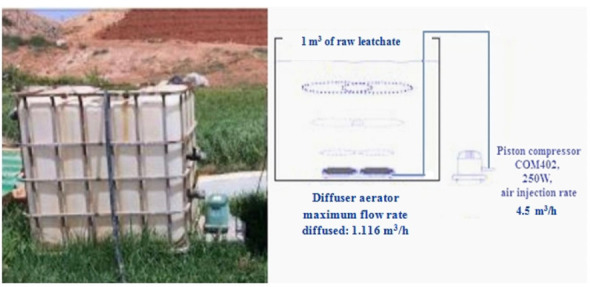
Aeration system of 1 m^3^

The intensive aeration technology, shown in Figure 4, was carried out in the site of the Mohammedia-Benslimane landfill. The raw leachate sample was randomly taken from the main storage tank and filled into a 1 m^3^ volume free surface polyethylene tank. The treatment of the discharge was carried out using an aeration technique ensuring the mechanical diffusion of air bubbles (installation of an air diffuser) which injected pressurized air at the bottom of the aeration basin with a flow rate of 18.6 L/min, corresponding to an oxygen quantity of 16.75 kg/m^3^/h, through a piston compressor with a maximum discharge capacity of 4.5 m^3^/h. The air insufflation system ensures a sufficient mixing of the mixture, homogeneous diffusion of air bubbles and the maintenance of a continuous agitation. The reactor operated at room temperature between 9°C and 12.7°C.

The duration of the aerobic treatment was 30 days, during which the purifying evolution was monitored by physico-chemical analyses. A weekly instantaneous sampling was carried out according to the French standard AFNOR,^11^ during the continuous treatment period.

## Methods of analysis

The main physico-chemical variables analyzed included electrical conductivity (EC), pH, chlorides (Cl-), chemical oxygen demand (COD), biological oxygen demand (BOD5), nitrates, ammonium, Kjeldhal nitrogen, suspended solids, phosphorus, and phenols.

All samples were analyzed for physico-chemical variables according to the procedure established in the standard method of the French Standards Association (FSA), Association Française de Normalisation (AFNOR).^11^ pH was measured using an “Accumet Basic AB15 pH meter”. Electrical conductivity was measured by the electrometric method, which is based on the measurement of the capacity of ions to transport the electrical current. This passage of electric current is carried out by the migration of the ions in a field produced by an alternating current. The measurement was performed using a conductivity meter (intelligent Conductivity pH meter YK-2001PH) according to the AFNOR standard 1994.^11^

Chemical oxygen demand was determined using the open reflux method (5220-B) according to the AFNOR standard 1994.^11^

The oxidation of the organic matter is carried out by sulphuric acid, silver sulphate and mercury sulphate acid. The sample was placed under reflux in a strongly acidic solution with a known excess of potassium dichromate. After 2 hours of digestion, the remaining potassium dichromate, which was not reduced, was titrated with ferrous ammonium sulfate. The Mohr salt determined the excess of potassium dichromate, according AFNOR standard 1994.^11^

Biological oxygen demand was evaluated by the respirometric method known as manometric, which automatically monitors the evolution of the BOD5 during the oxidation of organic matter. The method consists of filling a sample in hermetically sealed bottles of an appropriate size and incubating them at a determined temperature (20°C) for 5 days, according to the AFNOR standard 1994.^11^

The determination of suspended matter was carried out by the centrifugation method of the AFNOR standard 1994.^11^

Phenolic compounds were determined by the colorimetric method using the Folin-Ciocalteu reagent. The determination of nitrate in the sample was carried out by the spectrometric method in the presence of sulfosalicylic acid according to AFNOR standard 1994.^11^

The determination of total phosphorus was carried out by the spectrometric method according AFNOR standard 1994.^11^

The determination of ammoniacal nitrogen was carried out by the spectrophotometric method using indophenol blue according to the AFNOR standard 1994.^11^

Chlorides were measured by the Mohr method by titration of chloride ions. It consists of a silver determination of chloride ions by silver nitrate in the presence of sodium chromate. The latter is the colored indicator which reacts at the end of the dosage to form the silver chromate, appearing as a brick-red precipitate according to the AFNOR standard 1994.^11^

## Results

Table 2 shows the physico-chemical characteristics of the raw leachate taken from the storage tank. The pH value observed for the raw leachate *(Table 2)* during the present study was 7.62, representing a neutral pH. A high level of electrical conductivity of the leachate was represented by 48.4 ms/cm.

**Table 2:** Physico-chemical Characterization of Raw Leachate from the Mohammedia-Benslimane Landfill Site

**Parameters**	**pH**	**EC**	**COD**	**BOD5**	**SSM**	**NO_3_^-^**	**NH_4_^+^**	**TKN**	**Cl^-^**	**P**
**Unit**		mS/cm	mg O_2_/L	mg O_2_/L	mg/L	mg/L (N-NO3)	mg/L (NH4+)	mg/L (N)	mg/L	mg/L
**Concentration**	7.62	48.4	38600	24000	4620	33.14	4700	5932.45	6204.27	34

Abbreviations: EC, electrical conductivity; COD, chemical oxygen demand; BOD5, biological oxygen demand; SSM, solid suspended matter; NO_3_^-^, nitrate; NH_4_^+^, ammonium; TKN, total Kjeldahlnitrogen; Cl^-^; chlorine; P, phosphrous

Chemical oxygen demand and BOD5 showed high concentrations of 38600 mg/L and 24,000mg/L, respectively, with a biodegradability ratio BOD5/COD of 0.62. The leachate from the Mohammedia-Benslimane dump is a young type, with high NH_4+_ concentrations of about 4,700 mg/l and NTK and NO_3-_ concentrations of 5,932.45 mg/l and 33.14 mg/l, respectively. The concentration of chloride was 6204.27 mg/l and the phenolic compounds were measured at 0.11 mg/l.

### Biological treatment by intensive aeration

The results of the monitoring of the purifying evolution of the treated leachate are shown in Table 3. Over time, the pH increased from 7.62 to 9.46 at the end of treatment. Electrical conductivity concentrations ranged from 48.4 to 29.9 mS/cm *(Table 3).*

**Table 3:** Analysis of the Evolution of Raw and Treated Leachate

	Sampling campaigns*				

**Variables**	**C1**	**C2**	**C3**	**C4**	**C5**

	**LS**	**LA**	**LA**	**LA**	**LA**
COD (mg O2/L)	38600	22110	13440	10980	10520
BOD5 (mg O2/L)	24000	3000	1600	800	400
pH	7.62	8.92	9.36	9.37	9.46
EC (mS/cm)	48.4	36,6	33.7	27.9	29.9 w
SSM (mg/L)	4620	5700	7180	4000	6680
NO_3_^-^ (mg/L (N-NO3))	33.14	36.52	82.2	88.8	30.6
NH_4_^+^ (mg/L (NH4+))	4700	1490	2090	1630	720
TKN (mg/L (N))	5932.45	3275.41	2210.24	1924.7	1255.43
P (mg/L)	34	14	28	18	<0.5
Phenol (mg/L)	0.11	0.18	0.12	0.15	0.13
**Cl^-^** **(mg/L)**	6204.27	8508.72	10281.37	8508.72	9217.78

Abbreviations: LS, leachate from storage tank; LA, aerated leachate; COD, chemical oxygen demand; BOD5, biological oxygen demand; EC, electrical conductivity; SSM, solid suspended matter; NO_3_^-^, nitrate; NH_4_^+^, ammonium; TKN, total Kjeldahl nitrogen; P, phosphorus; Cl^-^, chlorine

^*^Sampling campaigns represent the samples taken from the landfill site each week (Cl is the first raw sample, C2 is the sample taken after one week of treatment, C3 is the sample taken after the second week of treatment, and C3 is the sample taken during the last week of treatment).

The results of the analyses performed showed that the COD and BOD5 values decreased over time. Chemical oxygen demand concentrations increased from 38,600 to 10,520 mg O_2_/L, and BOD5 concentrations increase from 24,000 to 400 mg O_2_/L *(Table 3).*

The nitrate content of the raw leachate was 33.14 mg/L. After biological treatment, this amount increased to 88.8 mg/L and then decreased to a concentration of 30.6 mg/L. Bacterial nitrification related to the mineralization of ammoniacal nitrogen to nitrate showed a decrease in ammoniacal nitrogen concentration from 4700 mg/L to 720 mg/L *(Table 3).*

### Purification efficiency

**Figure 5:**
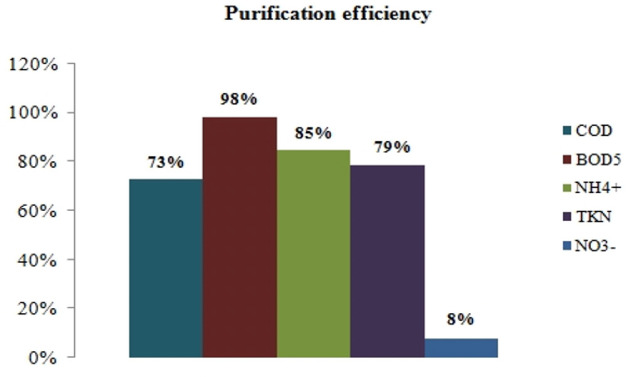
Chemical oxygen demand (COD), biological oxygen demand (BOD5), ammonium (NH_4+_), nitrate (NO_3_) and total Kjeldahl nitrogen (TKN) scrubbing efficiency

Figure 5 shows the purification yields of the aeration technique used.

The removal of BOD5 had the highest percentage with a 98% allowance, as does the removal of COD with a 73% allowance. Ammonium ions and Kjeldhal nitrogen had similarly high removal rates of 85% and 79%, respectively. The purification yield for nitrates was low at 8%.

## Discussion

According to Table 2, the hydrogen potential varied according to the age of the landfill, the nature of the waste being buried and the accumulation of volatile acid in the presence of methanogenic bacteria. Leachate from young landfills had a pH between 5.0 and 6.5, while leachate from old landfills had a pH between 7.8 and 8.64, Naveen *et al*. (2017) found the same pH value for leachates in India *(Table 4)*, and Mejraoua *et al*. (2017)found a basic pH value of about 8.99 for Meknes city landfill.^18,21^ This value falls within the defined range of old landfills, while Zalaghi *et al*. (2018) reported a pH value slightly lower than that of our previous case study for TAZA city landfill in Morocco *(Table 4)*.^15^ This can be explained by the similarity in the nature of the waste being discharged, and the climatic conditions of the region which favor the bacteriological and physico-chemical activity of the waste.

**Table 4—:** Comparison of Physico-chemical Variables of Raw Leachates

**Variables**	**pH**	**EC**	**COD**	**BOD5**	**Cl^-^**	**NO_3_^-^**	**NH_4_^+^**	**Ratio DBO5/COD**
**Unit**		mS/cm	mg/L	mg/L	mg/L	mg/L	mg/L	
**Raw leachate from the present study**	**7.62**	**48.4**	**38600**	**24000**	**6204.27**	**33.4**	**4700**	**0.62**
13	7.9		37.9	21.8	-	-	-	0.57
								
14	8.17	35	40000	10900	-	-	2540	0.28
**Tunisia**								
15	7.35	-	8245.4	5220	-	27.14	35.02	0.63
**Taza city landfill, Morocco**								
16	8.06	33.96	12626.74	5522.08	4289.51	-	3207.6	0.43
**Oum Azza landfill, Morocco**								
17	7.7	35.63	16160	2880	5731	137	2825	0.18
**Chandigarh site**								
18	7.6	3.87	12000	1500	780	28.00	2593	0.12
19	7.4	-	26183	17750	-	1318	-	0.68
20	6.653	-	57300	39500	-	2240	-	0.69
21	8.99	5.12	4808.1	157.18	-	14.17	44.86	0.03
**Meknes city landfill**								
22	7.95	26.3	12800	500	6381	480	1290	0.039
**Algeria**								
23	8.4	-	15225	5710	-	0.05	5208	0.38

Abbreviations: EC, electrical conductivity; COD, chemical oxygen demand; BOD5, biological oxygen demand; Cl^-^, chlorine; NO_3_^-^, nitrate; NH_4_^+^, ammonium

Electrical conductivity is a variable that gives an overall assessment of the concentration of ions present in the leachates, essentially of mineral types,^4^ and the concentration obtained in the present study indicated a strong mineralization of the raw leachates. The variation in EC is probably related to the concentrations of chloride ions present in the leachate. Ayoub *et al*. (2018) found a chloride concentration of 4,289.51mg/l and an electrical conductivity of 33.26 mS/cm for OumAzza landfill of Rabat city *(Table 4).*^16^ The value reported by Mor *et al*. (2018) of Cl- was 5731 mg/l for an EC of 35.63 mS/cm, and Naveen *et al*. (2017) reported a chloride ion concentration of 780 mg/l for an EC of the order of 3.87 mS/cm.^17,18^ It should be noted that the EC is proportional to the concentration of chloride ions *(Table 4).* In contrast, in a landfill in Algeria, a chloride value of 6381 mg/l and an EC of 26.3 mS/cm were found, indicating that the leachates were not sufficiently loaded compared to the leachates in the present study *(Table 4).*^22^

Chemical oxygen demand and BOD5 represent a high load of biodegradable organic matter. Leachate from a public landfill in Tunisia had a COD content higher than that of the present study with a value of 40,000 mg/l and a BOD5 content lower than that of the leachate in the present study with a value of about 10,900 mg/l, Arunbabu *et al.* (2017) reported higher leachate concentrations from an Indian landfill, with COD and BOD5 values of 57,300 mg/l and 39,500 mg/l, respectively, while Ogunlaja *et al*. (2018) presented low concentrations in terms of COD and BOD5 *(Table 4).*^14,13,20^ The measurement of COD and BOD5 content is an indicator of the effluent loading by biodegradable and non-biodegradable organic matter.

The BOD5/COD ratio is a good indicator of the biodegradability of the effluent. It has been shown that a BOD5/COD ratio value below 0.1 represents low biodegradability, while values above 0.5 represent very good biodegradability.^24^ The value obtained in the present study indicates that the leachates studied are young and have high biodegradability.

Leachates are loaded with trace elements such as ammonium, nitrogen, and chlorides. These elements are the main pollutants in leachates given their persistence in the environment and stability in anaerobic conditions. The accumulation of ammonium ion and nitrogen concentrations varies according to different phenomena such as the decomposition of proteins, hydrolysis, and the biodegradation of organic compounds by microorganisms.

Stabilized leachates generally have low biodegradability of organic content, with NH_4+_ and nitrogen concentrations as high as 1,426 mg/L.^17^ The NH_4+_, NTK, and NO_3-_ concentrations characterize the “acetogenic” phase of the leachates where hydrolysis, transformation and fermentation of the organic matter is done by the presence of bacteria producing simple and soluble molecules (fatty acids, ammonia).^25^ The variation in nitrate and ammonia nitrogen content can also be expressed by the natural biological oxidation of nitrogen. Mor *et al*. (2018) reported concentrations of NH_4+_ and NO_3-_, respectively, of 2,825 mg/l and 137 mg/l, while El-Gohary and Kamel. (2016) found concentrations of NH_4+_ and NO_3-_ of 5,208 mg/l and 0.05 mg/l, respectively *(Table 4).*^17,23^ According to the results obtained in the present study and in the literature, it was observed that the concentrations of nitrates and ammonium opposed each other (phenomenon of nitrification/denitrification).

The chemical composition of the leachate is specific to each landfill. It has a composition rich in organic matter such as humic and fulvic substances, volatile fatty acids, phenolic compounds, ammoniacal nitrogen, chlorinated organic compounds and toxic metals. According to the United States Environmental Protection Agency (USEPA), phenol is one of the main pollutants with serious harmful effects in humans expressed by anorexia, nausea, headaches, swallowing problems and fainting.^26^ While this substance is present in greater proportions in the leachate of Moroccan landfills compared to our previous case study.^4,27^ The composition of phenolic compounds in the leachate of the Mohammedia-Benslimane landfill greatly exceeded the acceptable concentration recommended by the World Health Organization (WHO) in drinking water of around 0.001 mg/l,^26^ but was below the Moroccan allowable discharge limit value of 0.5 mg/l.^10^

Characterization of the leachate from the Mohammedia-Benslimane landfill site showed the high biodegradability of the effluent which indicated the need for a biological treatment process as the bacteriological process is in an evolutionary phase. Biological treatment by intensive aeration is essentially based on the injection of a quantity of oxygen into raw leachate. To this end, the evolution of the chemical and biological processes is accelerated and consequently reduction of the pollutant load is promoted.

### Evolution of pH and EC

**Figure 6:**
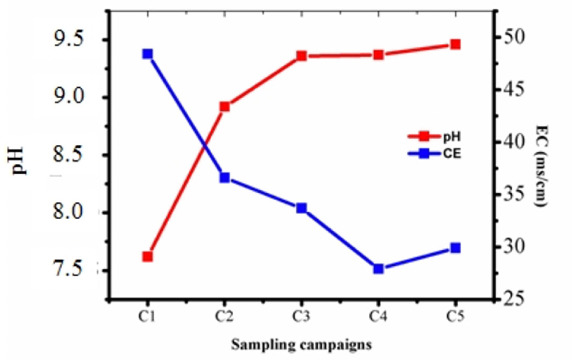
Effect of aeration on pH and electrical conductivity (EC)

Figure 6 shows the evolution of pH and electrical conductivity during the aerobic leachate treatment process. The increase in pH is probably due to the biological evolution through the consumption of volatile fatty acids (VFAs) that are metabolized by aerobic microorganisms.^28^

The decrease in conductivity is influenced, on the one hand, by the presence of microorganisms, which are naturally present in biomass leachates, indicating a strong correlation between electrical conductivity and biomass.^4^ On the other hand, the presence of high concentrations of ions has an effect on the change in conductivity, especially Cl-. According to Figure 5, the increase in pH coincided with a decrease in conductivity, and therefore aeration treatment stabilized the leachate to a stage where the biodegradability of the effluent became increasingly low.

### Effect on chemical and biological demand

**Figure 7:**
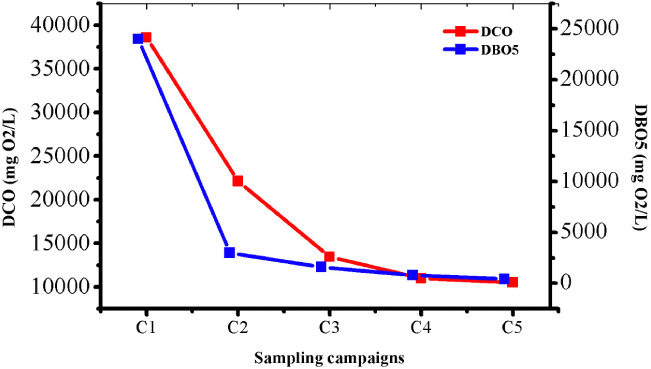
Effect of aeration on chemical oxygen demand (COD) and biological oxygen demand (BOD5)

Figure 7 shows the evolution of COD and BOD5 over the aerobic leachate treatment time. The injection of oxygen into the leachate facilitated the intensification of the bacterial activity which allowed the degradation of the organic matter, leading to a significant decrease in COD and BOD5 values *(Figure 7)*.^4^

The BOD5/COD ratio indicates the biodegradability of the leachate and this ratio decreases rapidly with the age of the landfill.^19^ This phenomenon is due to the release of large organic macromolecules, such as humic substances, into the environment.^29^ The average value of the BOD5/COD ratio for treated leachate is 0.09. The biodegradability becomes weaker and weaker over time and during treatment, as at the end of the treatment, the leachate becomes stable.

The observed reduction in pollution can also be explained by the activation of aerobic microorganisms consuming organic matter in the presence of sufficient oxygen, and the material present in the leachate is easily oxidizable and biodegradable. Biodegradability was also conditioned by the climate of the study site. It varied according to the seasons, as an increase in temperature favors the intensification of bacterial activity and therefore a more intensified degradation.^4^

### Effect of aeration on nitrates and ammonium

**Figure 8:**
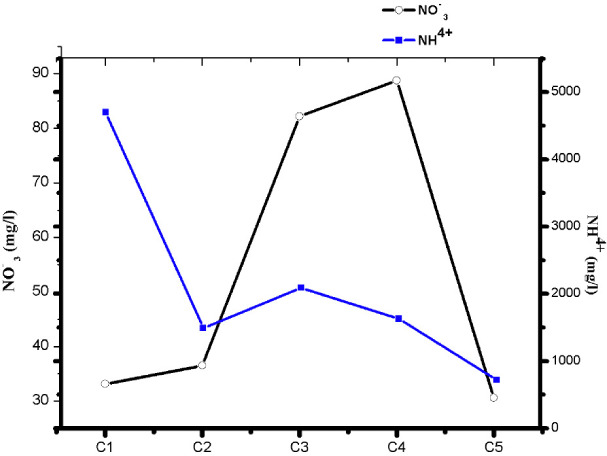
Effect of aeration on nitrification and denitrification

Figure 8 shows the effect of aeration on the nitrification/denitrification phenomenon. Biological nitrogen removal requires a two-step process involving aerobic nitrification of ammonia to nitrite *(Equation 1)* and subsequent conversion of nitrite to nitrate *(Equation 2)*, followed by anoxic denitrification denitrate/nitrite to nitrogen gas *(Equation 3).*^30^


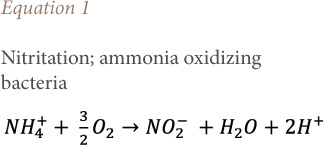



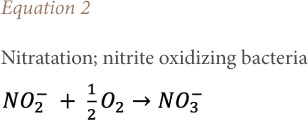



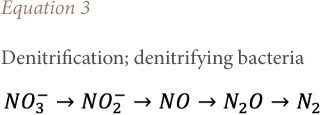


The variation in NH_4+_ and NO_3-_ concentrations highlights the nitrification/denitrification reactions. However, the treatment showed the nitrate removal cycle and a strong bacterial nitrification linked to the mineralization of ammoniacal nitrogen into nitrate which led to a decrease in the ammoniacal nitrogen concentration. Nag *et al.* (2019) reported the same concentrations of ammoniacal nitrogen.^31^ The intensive aeration treatment technique used gave satisfactory results in terms of purification and reduction of the pollutant load for certain pollutants contained in the leachate.

The removal of BOD5 and COD showed high abatement rates. Jirou *et al*. (2014) found higher abatement rates than in the present case study with removal percentages of 99.3% for COD and 99.1% for BOD5.^28^ Ammonium ions and Kjeldhal nitrogen had similarly high removal rates. The purification yield for nitrates was low with a value of 8%.

The removal rates found by Jirou *et al.* (2014) were higher than those found in the present case study due to the physico-chemical composition of the sample that was taken directly from the collection trucks (tipping trucks), with concentrations higher than those in the present study, indicating that it is fresh leachate, however the sample in the present study was taken from the leachate storage tank within the landfill.^28^

### Estimation of energy costs

The energy consumption of treatment technologies is a limiting factor in their use despite their high profitability in terms of efficiency and pollution removal (purification efficiency). Estimating energy consumption and the cost of leachate treatment by intensive aeration will make it possible to prove the efficiency of this technology and encourage its use by companies concerned with the treatment of leachates rich in biodegradable organic matter.

According to the present study, the leachate from the Mohammedia-Benslimane landfill showed a high load of biodegradable organic matter. Treatment by intensive aeration lasted 12 hours per day for 30 days. The electrical consumption of the pilot was as expressed in Equation 4:

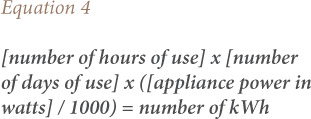



Calculation data: number of hours =12; number of days = 30; appliance power =250 watts.

Using the calculation formula yields:

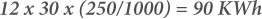



Management of the public service of the production and transport of electric energy as well as the distribution of electric energy is ensured by the national office of electricity and water in Morocco.The electric energy is sold, tariffs vary according to the bands of consumption, and the price in KWh is 1.5958 Dihrams (Dhs) for private electrification.^32^

The cost of consumption during the test period was calculated as 90 × 1.5958 = 143.9 Dhs.

The energy consumption costs of 90 KWh was 143.9 Dhs for the specific experimental conditions of a pilot of reduced size of 1 m^3^ of leachate and during 12 hours of continuous treatment for 30 days. The estimated cost of energy consumption varied according to the operating conditions of the treatment, including the quantity of leachate to be treated, the power of the compressors, the rate of oxygenation, and the type of leachate (young, intermediate, stabilized). The cost of energy consumption is the major limitation for the use of this technique.

## Conclusions

The results in the present study highlight the pollution generated by the Mohammedia-Benslimane landfill through production of leachates with a high pollution load. Characterization of the leachates identified their major characteristics, such as COD and BOD5, which showed high concentrations, and the BOD/COD ratio showed high biodegradability. Therefore, the discharge is of a young type and in a stage where biological activity is important.

The choice of treatment was based essentially on the composition of the leachate, which represented a high degree of biodegradability. The forced aeration biological treatment technique is based on intensive air injection into the leachate. This treatment considerably reduced a large part of the pollutant load and made it possible to achieve very high purification yields.The estimate of the cost of electrical energy consumption for leachate treatment by forced aeration varied according to the specific operating conditions of each experiment, depending on the operational context chosen.

## References

[1] Vaverková MD (2019). Landfill Impacts on the Environment — Review. Geosciences.

[2] (2017). World Bank Group M de l'environnement M. Le Coût de la Dégradation de l ' Environnement au Maroc. Environ Nat Resour Glob Pract Discuss.

[3] (2019). Rapport de synthèse, Département de l'Environnement, Ministère de l'Energies, des Mines de l'Eau et de l'Environnement. Strategie Nationale De Reduction Et De Valorisation Des Dechets. https://www.environnement.gov.ma/images/D%C3%A9chets/Rapport_de_synth%C3%A8se_SNRVD_FR.pdf.

[4] Abouri M, Souabi S, Elmaguiri A, Bakraouy H, Bahlaoui MA, Jada A (2016). Anaerobic-aerobic treatments of leachate from Municipal Solid Waste. International Conference on Advances in Civil Structural and Environmental Engineering ACSEE.

[5] Vallero DA, Blight G Waste (Second Edition): A Handbook for Management, Chapter 12 - The Municipal Landfill. Elsevier Inc 2019.

[6] Abdoul Razak MW, Adamou Z (2020). Caractérisation Physico-Chimique Des Lixiviats Des Décharges: Cas De La Décharge Non Contrôlée De Niamey 2000 (Niamey-Niger). Eur Sci J ESJ.

[7] Benyoucef F, El Ghmari A, Ouatmane A (2015). Etude expérimentale du traitement par évaporation forcée des lixiviats des déchets ménagers. Cas de la ville de Kasbah Tadla Déchets Sci Tech.

[8] Département de l'Environnement (2006). Loi n° 28-00 relative à la gestion des déchets et à leur élimination. https://www.environnement.gov.ma/images/Mde_PDFs/Fr/Actualisation_Cadre_Legislatif_05082016/6_Loi_28_00_relative_%C3%A0_la_Gestion_et_Elimination_des_D%C3%A9chets.pdf.

[9] Zhang L, Lavagnolo MC, Bai H, Pivato A, Raga R, Yue D (2019). Environmental and economic assessment of leachate concentrate treatment technologies using analytic hierarchy process. Resour Conserv Recycl.

[10] (2014). Valeurs limites de Rejet à respecter par les déversements (Normes de pollution). http://water.gov.ma/wp-content/uploads/2016/01/4.3.3.Valeurs-Limites-de-Rejet.pdf.

[11] (1994). Recueil de normes françaises 1994 Qualité de l'eau.

[12] Elmarkhi M, Sadek S, Elkharrim K, Benelharkati F, Belghyti D (2014). Contributions Methods Of Statistical Analysis Of Leachate From The Landfill. Int J Sci & Tech Res Vol 3 Issue 7.

[13] Ogunlaja A, Abarikwu SO, Otuechere CA, Oshoro OO (2019). Characterization of Leachates from Waste Landfill Sites in a Religious Camp along Lagos-Ibadan Expressway, Nigeria and Its Hepatotoxicity in Rats. Chemosphere.

[14] Smaoui Y, Bouzid J (2019). Déchets du centre d'enfouissement technique de Sfax (Tunisie) : nature, composition et traitement. Déchets Sci Tech.

[15] Zalaghi A, Lamchouri F, Merzouki M, Toufik H (2018). Treatment by the process sequencing batch reactor (SBR) of leachates from the uncontrolled public landfill in the city of Taza (Morocco). Traitement par le procédé SBR ( Sequencing Batch Reactor ) des lixiviats de la décharge publique non contrôlée de. Int J Innov Appl Stud.

[16] Ayoub EL-A, Naim E, Aziz EL-B, Mohamed S, Safae T, Ahmed EL-B, Khadija EL-K, Driss B (2018). Study of the impact of Oum Azza landfill leachates on the environment of Rabat - Morocco. Int J of Environ & Agri Research (IJOEAR).

[17] Mor S, Negi P, Khaiwal R (2018). Assessment of groundwater pollution by landfills in India using leachate pollution index and estimation of error. Environ Nanotechnology Monit Manag.

[18] Naveen BP, Mahapatra DM, Sitharam TG, Sivapullaiah P V, Ramachandra T V (2017). Physico-chemical and biological characterization of urban municipal landfill leachate. Environ Pollut.

[19] Alver A, Altaş L (2017). Characterization and electrocoagulative treatment of landfill leachates: A statistical approach. Process Saf Environ Prot.

[20] Arunbabu V, Indu KS, Ramasamy E V (2017). Leachate pollution index as an effective tool in determining the phytotoxicity of municipal solid waste leachate. Waste Manag.

[21] Mejraoua Z, Zine N-E (2017). Caracterisation Physico-Chimique Du Lixiviat De La Decharge Sauvage De Meknes. Euro Sci J.

[22] Oussama L, Mohammed B (2017). Les risques de la pollution du milieu naturel par les lixiviats des décharges contrôlées. Cas du centre d'enfouissement technique de Maghnia.

[23] El-Gohary FA, Kamel G (2016). Characterization and biological treatment of pre-treated landfill leachate. Ecol Eng.

[24] Arij Y, Fatihah S, Rakmi AR (2018). Performance of pilot scale anaerobic biofilm digester (ABD) for the treatment of leachate from a municipal waste transfer station. Bioresour Technol.

[25] Souhaila T Études de traitement des lixiviats des déchets urbains par les Procédés d'Oxydation Avancée photochimiques et électrochimiques. Application aux lixiviats de la décharge tunisienne “Jebel Chakir”.2012. https://tel.archives-ouvertes.fr/tel-00674731.

[26] Ahmadinejad SO, Naeeni STO, Akbari Z, Nazif S Investigating the performance of agricultural wastes and their ashes in removing phenol from leachate in a fixed-bed column. Water Sci Technol.

[27] Bakraouy H, Souabi S, Digua K, Dkhissi O, Sabar M, Fadil M (2017). Optimization of the treatment of an anaerobic pretreated landfill leachate by a coagulation–flocculation process using experimental design methodology. Process Saf Environ Prot.

[28] Jirou Y, Harrouni MC, Belattar M, Fatmi M, Daoud S (2014). Traitement des lixiviats de la décharge contrôlée du Grand Agadir par aération intensive. Rev Mar Sci Agron Vét.

[29] Moulin P (2013). Lixiviat de centre de stockage : déchet généré par des déchets. http://www.revue-ein.com.

[30] Saraiva I, Sousa MA, Gonc C (2013). Multistage treatment system for raw leachate from sanitary landfill combining biological nitrification-denitrification/solar photo-Fenton/biological processes, at a scale close to industrial--biodegradability enhancement and evolution profile of trace pollutants. Water Res.

[31] Nag M, Shimaoka T, Komiya T (2018). Influence of operations on leachate characteristics in the Aerobic-Anaerobic Landfill Method. Waste Manag.

[32] National Office for Electricity and Drinking Water (NOEDW) Electricity branch. http://www.one.org.ma/FR/pages/interne.asp?esp=1&id1=3&id2=113&t2=1.

